# Two successful pregnancies ‐in patients taking Volanesorsen for familial chylomicronemia syndrome

**DOI:** 10.1002/jmd2.12435

**Published:** 2024-06-16

**Authors:** Subadra Wanninayake, Antonio Ochoa‐Ferraro, Karishma Patel, Radha Ramachandran, Anthony S. Wierzbicki, Charlotte Dawson

**Affiliations:** ^1^ Department of Diabetes, Endocrinology and Metabolism University Hospitals Birmingham NHS Foundation Trust Birmingham UK; ^2^ The Grantham Practice London UK; ^3^ Department of Adult Inherited Metabolic Diseases, Metabolic Medicine and Chemical Pathology Guys and St Thomas' Hospitals NHS Foundation Trust London UK

**Keywords:** familial chylomicronemia syndrome (FCS), hypertriglyceridemia, pregnancy, Volanesorsen

## Abstract

Familial chylomicronemia syndrome (FCS) is a rare inherited disorder characterized by severe hypertriglyceridemia, posing a heightened risk of acute pancreatitis. Recently, Volanesorsen, an APOC3 antisense oligonucleotide, gained approval for FCS treatment in the UK. Caution is advised during pregnancy due to limited safety data, although animal studies show no toxicity/teratogenicity. Two case scenarios are presented: In the first case, a patient with FCS continued Volanesorsen injections without having thrombocytopenia during an unplanned pregnancy until third trimester, maintaining triglyceride control. Upon discovering the pregnancy at 38 weeks, Volanesorsen was ceased, and a low‐fat diet reinstated. Despite a heightened risk of pancreatitis, no episodes of pancreatitis occurred during the pregnancy. In the second case, stopping Volanesorsen before conception led to elevated triglycerides, and an episode of acute pancreatitis at 22 weeks, despite strict very low‐fat diet and fibrate therapy from 14 weeks. At 23 weeks, Volanesorsen was reintroduced concurrently with regular therapeutic plasma exchange. No further episodes of pancreatitis occurred. In both case, fetal health was maintained throughout pregnancy, fetal scans revealed no anomalies, and planned C‐sections delivered healthy babies without congenital issues. Both babies are well and developing normally at 24 and 19 months.

**Synopsis**

This paper describes successful pregnancies for two females with FCS whilst taking volanesorsen. Neither patient experienced pancreatitis when taking volanesorsen and both gave birth to health babies at term.

## 1 BACKGROUND

Familial chylomicronemia syndrome (FCS, OMIM #238600) is a rare autosomal recessive disorder resulting from the deficiency of lipoprotein lipase (LPL, 8p21.3), apoprotein C2 (APOC2, 19q13.32), apolipoprotein A5 (APOA5, 11q23.3), lipase maturation factor 1 (LMF1, 16p13.3), glycosylphosphatidylinositol‐anchored‐high‐density lipoprotein‐binding protein 1 (GPIHBP1, 8q24.3), and glycerol‐3‐phosphate dehydrogenase 1 (GPD1, 12q13.12), with additional genes expected to be identified in the future.^1^

Lipoprotein lipase (LPL), an enzyme present in the capillary endothelium of adipose and muscle tissue, plays a crucial role in triglyceride (TG) lipolysis in chylomicrons and other triglyceride‐rich lipoproteins (TGRL).^2^ LPL activity is regulated by several factors, including APOC2, an essential cofactor for LPL activity, APOA5 which stabilizes the LPL‐TGRL complex, and APOC3 which inhibits LPL activity. FCS manifests as severe hypertriglyceridemia (TG ≥10 mmol/L [885 mg/dL]), resulting in a milky appearance in plasma^1^, ^3^ with a concurrently elevated TG/total cholesterol ratio (>2 mmol/mmol [>5 mg/mg]) and low apolipoprotein B concentration (<100 mg/dL). Acute hypertriglyceridaemic pancreatitis is the most serious complication of FCS. Recurrent acute episodes are associated with poor clinical outcomes including exocrine and endocrine pancreatic insufficiency leading to malabsorption and diabetes respectively.^1^, ^2^

Management of FCS involves a highly restrictive low‐fat diet (less than 20 g of fat daily for adults) and lipid‐lowering agents. Adherence is often challenging. Traditional lipid‐lowering drugs such as statins and fibrates exhibit limited efficacy. Even with optimal adherence to low‐fat diet and continued fibrates, pregnant individuals with FCS face a heightened risk of pancreatitis.^4^, ^5^ The recent approval of the novel APOC3 antisense oligonucleotide, Volanesorsen, has demonstrated a significant reduction in triglycerides (77% vs. 18% in the control group, APPROACH trial).^6^ While caution is advised due to limited safety data in pregnancy, animal studies have not indicated toxicity or teratogenicity. Here, we present two case scenarios: an undetected pregnancy in a patient on Volanesorsen who successfully delivered at −38 weeks of gestation and a planned pregnancy in a patient where Volanesorsen was reintroduced from mid‐gestation.

## 2 CASE 1

A 22‐year‐old female with FCS (compound heterozygous for two *LPL* variants) was diagnosed at the age of 6 months during an investigation for failure to thrive and elevated triglycerides (>70 mmol/L). The patient received adequate management with Monogen feeds supplemented with essential fatty acids and fat‐soluble vitamins throughout childhood. However, her teenage years were marked by recurrent hypertriglyceridaemic pancreatitis episodes (TG >20 mmol/L), mistakenly attributed to gallstones leading to a cholecystectomy.

**FIGURE 1:**
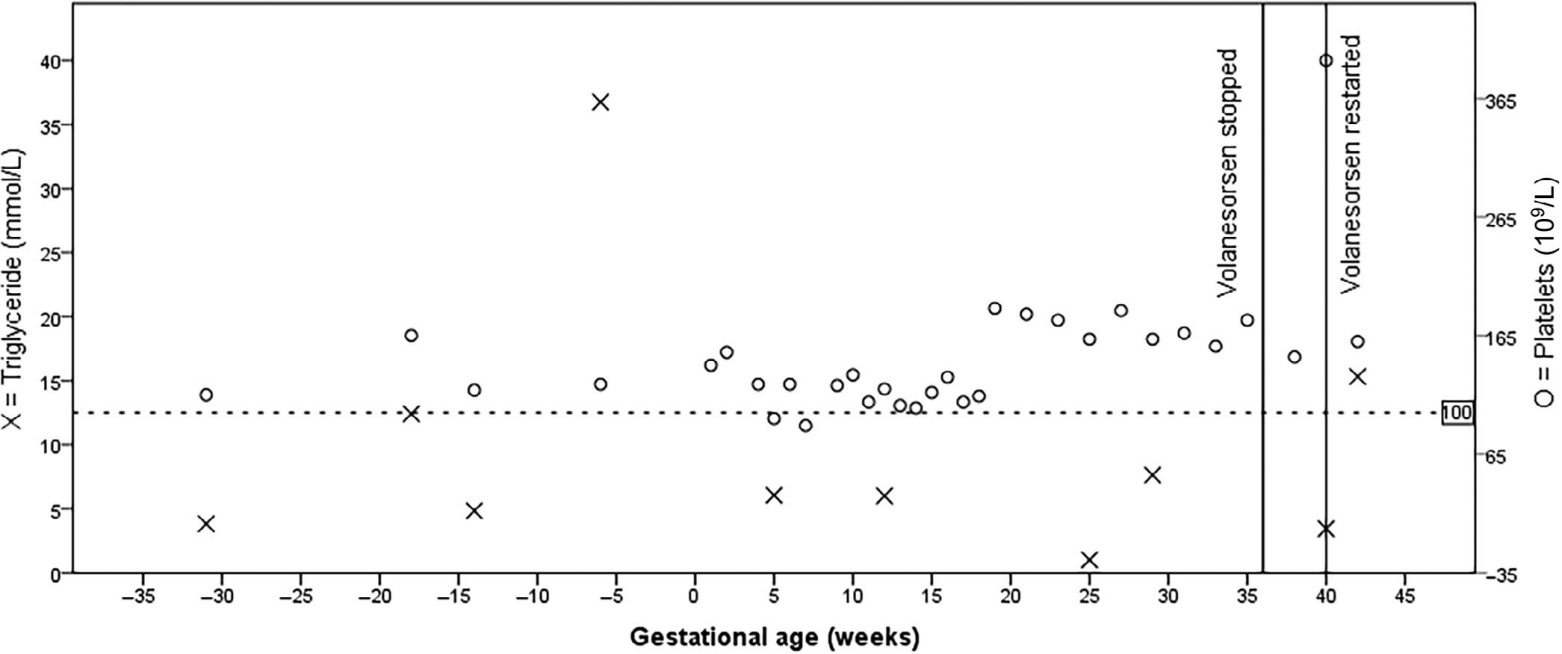
Case 1—Changing pattern of triglycerides and platelet count during pregnancy.

From the age of 18, Volanesorsen 285 mg was administered fortnightly as part of the Early Access to Medicines Scheme (EAMS) and subsequently continued following the National Institute for Health and Care Excellence (NICE) Highly Specialized Technology guidance 13 (HST13). Treatment continuity was only interrupted during periods of thrombocytopenia, closely monitored when platelets fell below 100 × 10^9^/L (Figure 1).

While on Volanesorsen, the patient did not require hospitalizations for acute pancreatitis, successfully completing her studies and commencing work in the catering sector. At aged 21, at an estimated gestational age (EGA) of 32 weeks, the patient discovered she was pregnant. Due to limited safety data on Volanesorsen use during pregnancy, a mutual decision was made to discontinue it, opting for a strict, very low‐fat diet (<10 g/day) supplemented with MCT oil. However, a subsequent fetal ultrasound revealed she was actually at 38 weeks gestation, with the fetus in a transverse position, prompting a planned cesarean section at 39 weeks. A healthy baby boy weighing 3.4 kg was born without structural defects. The patient resumed Volanesorsen treatment post‐delivery after 2 weeks of off treatment and utilized formula milk for newborn feeding. Notably, the patient did not experience any episodes of acute pancreatitis during her pregnancy. To date the 24 months‐old toddler is showing normal development and meeting all milestones.

## 3 CASE 2

A female with FCS (double heterozygous *GP1HBP1* and *APOA5* variants) diagnosed at birth following the discovery of markedly elevated triglyceride levels of approximately 200 mmol/L. She managed her condition with a very low‐fat diet and supplemented with essential fatty acids and fat‐soluble vitamins until adolescence. However, during her early 20s, she experienced recurrent episodes of hypertriglyceridemic pancreatitis, characterized by triglyceride levels well in excess of 20 mmol/L.

She was managed with a fortnightly dose of Volanesorsen 285 mg for a continuous period of 5 years, with regular monitoring for thrombocytopenia with dosing interval adjusted according to established protocol. With this treatment regimen, she was able to graduate and achieved remarkable success in both her professional and personal life, all while avoiding any further episodes of pancreatitis.

**FIGURE 2:**
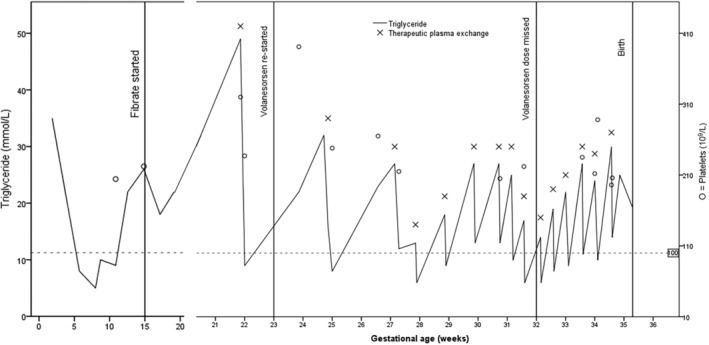
Case 2—Changing pattern of triglycerides and platelet count during pregnancy.

Due to a lack of safety data, and in line with current guidance, approximately 6 months before conception, Volanesorsen was discontinued, resulting in an increase in her triglyceride levels. By the end of the 14th week of gestation, fibrate therapy was initiated however, in the 22nd week of pregnancy (Figure 2), she experienced an acute pancreatitis episode. Subsequently, Volanesorsen was reintroduced in the 23rd week of pregnancy, after obtaining clinical governance approval^7^ and in addition, therapeutic plasma exchange (TPE) was initiated (initially fortnightly and then progressing to twice weekly) to maintain safe triglyceride levels. She had no further in‐patient admissions with pancreatitis and successfully gave birth to a healthy baby at a gestational age of 35 weeks.

## 4 DISCUSSION

Prior to the availability of Volanesorsen, treatment for FCS, as in our patients, was ineffective comprising a highly restrictive poorly tolerated low‐fat diet, fibrates, omega‐3 fatty acids (2–4 g/day) and medium chain triglyceride oil.^1^, ^8^

Volanesorsen is a second‐generation antisense oligonucleotide to the LPL inhibitor molecule ApoC3. It is administered via subcutaneous injection and functions by promoting lipoprotein lipase (LPL) activity and encouraging an LPL‐independent pathway for chylomicron metabolism.^9^

The inclusion of pregnant women in randomized controlled trials is limited by medical, legal, and ethical barriers and information about the safety and efficacy of medications in pregnancy relies on post‐marketing surveillance alongside the use of animal models. It's noteworthy that clinical trials focusing on the use of pharmaceutical drugs in pregnant women constitute less than 0.5% of registered clinical trials.^10^, ^11^, ^12^, ^13^

During pregnancy, significant anatomical and physiological changes occur. Left ventricular hypertrophy and increased plasma volume, peaking at 32 weeks, are adaptations to meet the elevated demands of the placenta and fetus. Simultaneously, drug elimination is influenced by changes in liver enzyme activity, hepatic blood flow, and an elevated glomerular filtration rate (GFR) throughout pregnancy, resulting in alterations in drug levels in maternal blood.^10^, ^11^, ^12^, ^13^, ^14^, ^15^ As a consequence, there is a decrease in plasma albumin and a slightly reduced α1‐glycoprotein, leading to higher concentrations of free drug for protein‐bound medications. As volanesorsen is >97% protein bound in plasma, free drug concentration could be significantly elevated.^16^ Patient characteristics, gestational timing, comorbidities, and concurrent medications also impact drug pharmacokinetics.^13^ Although intact volanesorsen is freely excreted through the kidney, the concentration of both volanesorsen and its metabolites (N‐1 to N‐3 metabolites and N‐5 to N‐14) are accumulated in both liver and kidney for a considerable time following the dose, thus it highlights the importance of considering dose, efficacy and side effect of the volanesorsen during pregnancy.^16^

The use of medications during pregnancy poses potential risks, including abortion, fetal growth restriction, preterm delivery, teratogenicity, fetal toxicities, and long‐term effects on the child's health.^11^ Placental transport is influenced by factors such as protein binding, ionization degree, lipid solubility, and molecular weight. The placental membrane allows the simple diffusion of small (<500 Da), lipid‐soluble, ionized, and poorly protein‐bound molecules.^13^ Selective transportation across the placenta serves to shield the fetus from exposure to harmful xenobiotics, safeguarding against adverse effects.^15^ The expression of transporters in the placenta is regulated by various factors, including hormones (oestradiol, progesterone, and glucocorticoids), diseases (inflammation, infection, and cancer), cellular factors (growth hormone, cytokines), and genetic polymorphisms.^13^, ^17^ Registries are being developed to track the effects of off‐label medications that have been required for specific disease indications during pregnancy.^18^

Although there is no study to evaluate the impact of volanesorsen on the human fetus, pre‐clinical studies in mice and rabbits showed relatively low concentrations in placental tissue and no measurable levels in fetal tissue despite the paternal and maternal hepatic tissue concentrations of intact volanesorsen being increased in a dose‐dependent manner. This may indicate that volanesorsen is not readily transported to the embryo or fetus. Decreases in fetal body weight were observed which probably secondary to decreased maternal body weight and food consumption, rather than a direct effect of volanesorsen on fetal development.^19^ Fortunately, our two cases did not show any developmental anomalies particularly, despite case 1 having been on volanesorsen throughout most of her pregnancy.

Hypertriglyceridemia‐induced acute severe pancreatitis is a well‐known complication in patients with FCS.^20^ While the exact mechanism is not entirely understood, it is generally accepted that chylomicrons, triglyceride‐rich lipoprotein large particles present in circulation when triglycerides exceed 10 mmol/L (885 mg/dL), obstruct pancreatic capillaries, leading to ischemia and acidosis. Subsequent acinar cell injury releases pancreatic lipase, enhancing lipolysis and resulting in an increased concentration of free fatty acids (FFA) in the pancreas. FFAs release inflammatory mediators and free radicals, inducing inflammation, oedema, and necrosis while chylomicrons are associated with macrophage and neutrophil activation.^21^, ^22^

Acute pancreatitis in normal pregnancy has an incidence of 1/1000–10 000, with most cases occurring in the third trimester or early postpartum, indicating a 2‐4‐fold increased risk of pancreatitis during this period. As in non‐pregnant individuals, the commonest causes are gallstone disease, alcohol excess, and hypertriglyceridemia. However, hypertriglyceridemia‐induced pancreatitis accounts for a relatively higher proportion of cases in pregnancy compared with the non‐pregnant population (56% vs. 1%–35%).^23^, ^24^ This may be because of a physiological reduction in lipoprotein lipase (LPL) activity during the third trimester,^4^, ^20^ which has profound implications for individuals with LPL deficiency leading to an exceptionally high risk of pancreatitis in the third trimester and peripartum.^25^

Pregnancy‐associated acute pancreatitis mimics other conditions presenting with abdominal pain and mimics the onset of labor, leading to delayed or missed diagnosis with poorer outcome. Hypertriglyceridemia‐induced pancreatitis is associated with a more severe course of disease and worse clinical outcomes compared to pancreatitis caused by other disorders, potentially leading to permanent pancreatic tissue damage, pancreatic insufficiency, and type 3 diabetes.^1^, ^2^, ^3^, ^8^ Hence FCS is associated with an increased risk of maternal and fetal morbidity and mortality during pregnancy.

Our first case did not experience any episodes of acute pancreatitis during her pregnancy and had an uneventful full‐term pregnancy with delivery of a healthy baby. She remained on Volanesorsen throughout pregnancy up until the penultimate week when the pregnancy was discovered. Triglycerides were not monitored frequently throughout this pregnancy because it was concealed until a late stage. On the other hand, our second case experienced pancreatitis during the second trimester while off Volanesorsen in line with suggested licensing indications. Volanesorsen was restarted at 23 weeks. Given the risks associated with hypertriglyceridemia in pregnancy, and the time‐lag associated with volanesorsen reaching a steady state that offers effective triglyceride lowering, TPE was concurrently started as a precaution. We cannot know whether plasmapheresis could have been avoided or deferred had she been on Volanesorsen throughout pregnancy. Furthermore, the bioavailability and impact on circulating levels of Volanesorsen while on regular therapeutic plasma exchange is not known. However, Volanesorsen from week 23 did not have any adverse outcome for the baby.

In summary, FCS is an important cause of significant hypertriglyceridemia that can lead to recurrent episodes of acute severe pancreatitis. Pregnancy is a time of exceptional risk of this complication. Volanesorsen is the first treatment available which prevents pancreatitis in FCS but its safety in pregnancy was unknown. To our knowledge, these are the first two pregnancies reported while on Volanesorsen, both with successful outcome for the mother and baby. This treatment could present FCS patients an option to potentially control their TG levels during pregnancy and reduce, or eliminate, the need for admission to treat acute pancreatitis or administer regular plasmapheresis.

## AUTHOR CONTRIBUTIONS

CD, AO, and SW conceived the study. AO, SW, and KP were involved in data collection and drafted the manuscript. All authors made contributions to writing and reviewing of the final manuscript.

## FUNDING INFORMATION

No grants or fellowships have supported the writing of this paper.

## CONFLICT OF INTEREST STATEMENT

Antonio Ochoa‐Ferraro received consultant fees from AKCEA and SOBI and donated to the University Hospital Birmingham charity in full. Other authors have no conflict of interest to declare.

## INFORMED CONSENT

Written informed consent was obtained from the patients for their anonymized information to be published in this article.

## Data Availability

Anonymised data presented in this manuscript are available for review upon reasonable request.
